# Worry Postponement From the Metacognitive Perspective: A Randomized Waitlist-Controlled Trial

**DOI:** 10.32872/cpe.12741

**Published:** 2024-06-28

**Authors:** Clara Krzikalla, Ulrike Buhlmann, Janina Schug, Ina Kopei, Alexander L. Gerlach, Philipp Doebler, Nexhmedin Morina, Tanja Andor

**Affiliations:** 1 Institute of Psychology, University of Münster, Münster, Germany; 2 Department of Psychology, Institute of Clinical Psychology and Psychotherapy, University of Cologne, Cologne, Germany; 3 Department of Statistics, Technical University of Dortmund, Dortmund, Germany; Philipps-University of Marburg, Marburg, Germany

**Keywords:** worry postponement, metacognitive therapy, generalized anxiety disorder, hypochondriasis, stand-alone-intervention

## Abstract

**Background:**

Pathological worry is associated with appraisals of worrying as uncontrollable. Worry postponement (WP) with a stimulus control rationale appears to be effective in non-clinical samples. However, preliminary research in participants with generalized anxiety disorder (GAD) does not support its efficacy in reducing negative metacognitions or worry. The aim of this study was to investigate the efficacy of WP with a metacognitive rationale.

**Method:**

Participants with GAD (n = 47) or hypochondriasis (HYP; n = 35) were randomly assigned to either an intervention group (IG) or waitlist (WL). The IG received a two-session long WP intervention aiming at mainly reducing negative metacognitions concerning uncontrollability of worrying. Participants were instructed to postpone their worry process to a predetermined later time during the six days between the two sessions. Participants completed questionnaires of negative metacognitions and worry at pre-assessment, post-assessment, and follow-up.

**Results:**

We observed a significant Time*Group interaction for negative metacognitions and worry. Post-hoc analyses on the total sample and separately for GAD and HYP revealed significantly lower worry scores in the treated GAD sample compared to the WL, representing the only significant effect. In the GAD group, pre-post-effect sizes were small for negative metacognitions and large for worry. Effects persisted to a four-week follow-up.

**Conclusion:**

WP with a metacognitive rationale seems to be effective in reducing worry in participants with GAD. The effectiveness for HYP seems limited, possibly due to the small sample size.

## Highlights

- Negative metacognitions about the uncontrollability of worrying maintain pathological worry.
- WP reduced negative metacognitions in participants with generalized anxiety disorder (GAD).
- WP further reduced worry in participants with GAD, with a recovery rate of 40%.
- Evidence for efficacy of WP in hypochondriasis is limited.

Excessive and uncontrollable worry represents the core symptom of generalized anxiety disorder (GAD) and it also plays an important role in other mental disorders, including hypochondriasis (HYP; Chelminski & Zimmerman, 2003; Ehring & Behar, 2020; Jansson-Fröjmark et al., 2020). Metacognitive theory for GAD (Wells, 2009) defines worrying as a coping strategy triggered by intrusive negative thoughts about potential future events. The development and perpetuation of GAD depend on the emergence of negative metacognitions concerning the uncontrollability of worrying, or its detrimental and perilous consequences. Previous research supports the influence of negative metacognitions on GAD symptoms (e.g., Nordahl et al., 2023; Penney et al., 2013; Wells, 2010). Metacognitive therapy (MCT) addresses negative metacognitions using techniques such as attention training (ATT), detached mindfulness (DM), and worry postponement (WP). While the overall effectiveness of MCT has been demonstrated in a wide range of disorders (Normann & Morina, 2018; Sharma et al., 2022), investigating individual components of the treatment allows for a better understanding of its mechanism and therapeutic potential. Distinguishing between active and inactive treatment components can improve treatment efficacy, patient retention, and treatment dissemination. ATT and DM are effective even when applied as stand-alone-interventions (Gkika & Wells, 2015; Knowles et al., 2016; Rochat et al., 2018; Rupp et al., 2019). Further research on the efficacy of WP is needed.

In WP with a metacognitive rationale, patients are instructed to challenge their uncontrollability beliefs by consciously delaying worrisome thoughts to a predefined later time, rather than engaging with them whenever they arise (Wells, 2006). This should increase patients’ control over the worry process and enable more functional metacognitions and behaviors. Historically, WP has been employed in cognitive behavioral therapy with a stimulus control rationale. Borkovec et al. (1983) noted that worry can become associated with a variety of stimuli. Striving to reduce stimulus generalization and uncontrollable worry, patients are asked to postpone their worry to a predefined worry period at the same time and location every day. This approach has shown promising results with effects particularly on worry duration in kids and psychology students (Borkovec et al., 1983; Brosschot & van der Doef, 2006; Jellesma et al., 2009; McGowan & Behar, 2013; Verkuil et al., 2011). However, in a study with GAD patients (Tallon, 2019), WP showed no effect on worry or metacognitions compared to control conditions. None of these studies explicitly addressed negative metacognitions. Consequently, the efficacy of WP with a metacognitive rationale remains unknown.

Against this background, we aimed to assess the efficacy of WP with a metacognitive rationale in its original target population – patients with GAD. As MCT is considered a transdiagnostic treatment (Wells, 2009), we included another clinical sample – patients with HYP – to test if this transdiagnostic property of MCT applies to WP. In HYP, illness worries and preoccupation with fears of having a serious disease are common symptoms (American Psychiatric Association, 2013; Fink et al., 2004; Noyes, 1999). Previous research suggests that metacognitions, in particular the belief of uncontrollability, may also play an important role in health anxiety (Bailey & Wells, 2016; Melli et al., 2018). There is some preliminary evidence that MCT and/or ATT may be helpful in treating HYP (Bailey & Wells, 2014; Papageorgiou & Wells, 1998). Therefore, we opted to include this patient sample in our study.

Based on these findings, we hypothesized that WP would reduce negative metacognitions and worry in both GAD and HYP from pre- to post-assessment compared to a waitlist control group. Furthermore, we anticipated a sustained reduction in symptoms from pre-assessment to the 4-week follow-up assessment for the clinical samples.

## Method

### Recruitment

This randomized wait-list controlled trial was conducted between 2011 and 2014 at the psychotherapy outpatient clinic at the University of Münster. It was approved by the Institutional Review Board of the Department of Psychology and Sport Science at the University of Münster.

Participants were recruited via newspaper advertisements, brochures in medical practices, the website of the specialized unit for the treatment of GAD of the psychotherapy outpatient treatment center at the University of Münster, and by informing patients during consultation appointments in the psychotherapy outpatient treatment center. Inclusion criteria were: diagnosis of GAD as assessed with the Structured Clinical Interview for DSM-IV Axis I Disorders (Wittchen et al., 1997) or HYP according to the criteria proposed by Fink et al. (2004), age between 18 and 65 years, and sufficient German language skills. Due to criticism of the strict DSM-IV criteria for HYP (Weck et al., 2014) and the fact that the DSM-5 (American Psychiatric Association, 2013) was not yet available, we used the preliminary criteria of Fink et al. (2004). This is also the reason why we use the term hypochondriasis throughout the manuscript, as it reflects the diagnostic criteria used at the time of data collection. Fink et al. (2004) emphasized uncontrollability, as their first criterion is obsessive rumination with intrusive thoughts, ideas, or fears of harboring an illness that cannot be stopped or can be stopped only with great difficulty. In contrast to DSM-IV (American Psychiatric Association, 1994), both Fink et al. (2004) and the DSM-5 (American Psychiatric Association, 2013) dropped the criterion that the preoccupation with a serious illness persists despite appropriate medical evaluation and reassurance. However, the DSM-5 (American Psychiatric Association, 2013) requires six months of symptom duration, whereas Fink et al. (2004) require only two weeks. Exclusion criteria were: current psychotherapy, a DSM-IV diagnosis of alcohol or substance abuse, psychotic symptoms, active suicidal ideation, or any change in psychotropic medications within the last three months.

### Procedure

**Figure 1:**
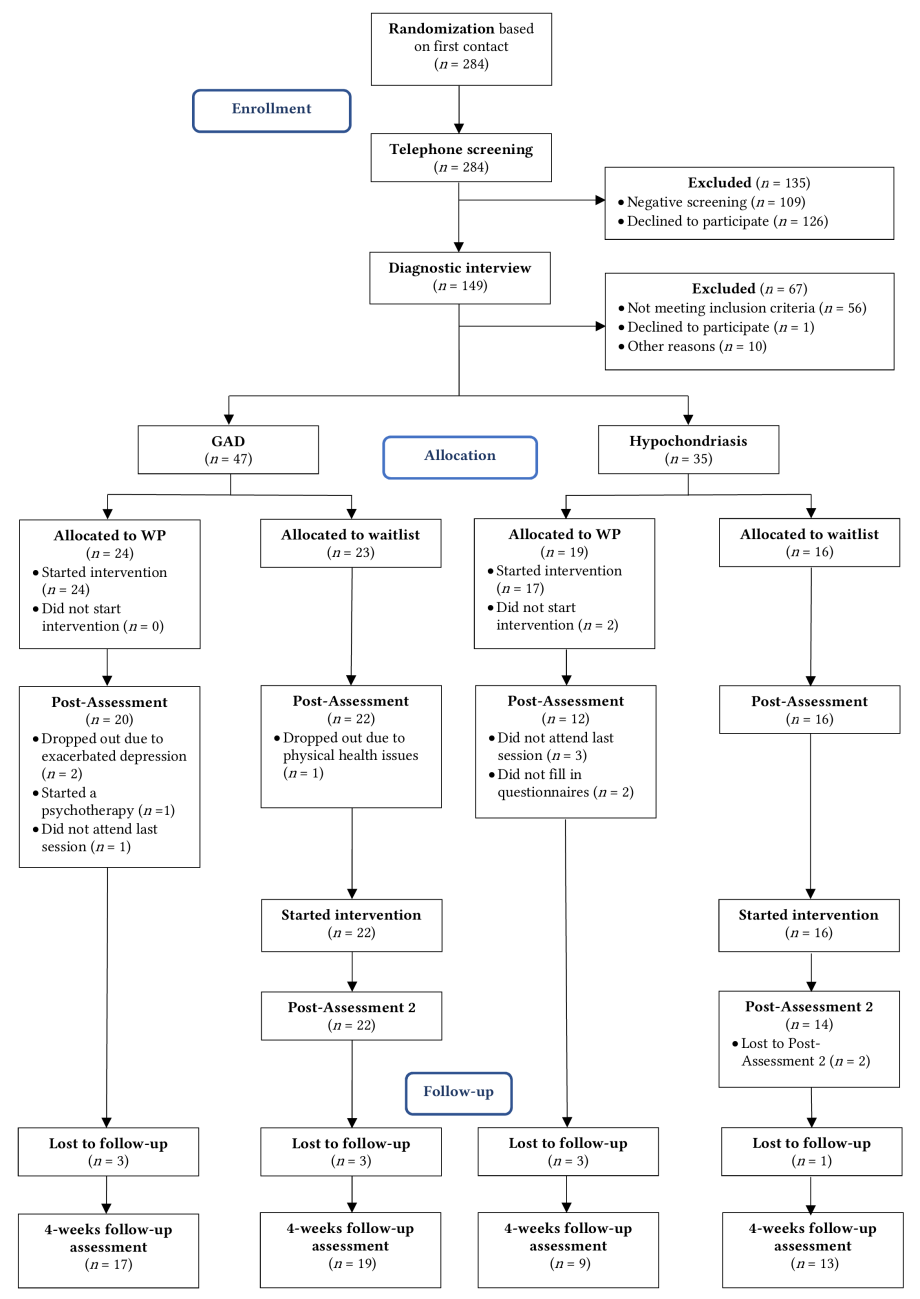
CONSORT Flow Diagram *Note.* GAD = Generalized Anxiety Disorder; WP = Worry Postponement.

Participants were randomized using simple randomization to either an intervention group (IG) or waitlist control group (WL) when first contacting the outpatient treatment center. The diagnostic criteria were initially assessed through a telephone screening. Subsequently, another investigator performed a detailed diagnostic examination face to face. Participants in the IG received a two-session-long WP intervention based on the metacognitive model of GAD one week after the diagnostic session. The WL also received the intervention after post-assessment. Data were collected prior and one week after the intervention/waiting period. Participants received a short telephone session with information about further treatment options, if necessary, after returning the questionnaires. Part of the post-assessment data collection were also ecological momentary assessment data over one week. These were published elsewhere (Thielsch et al., 2015). All participants received follow-up (FU) questionnaires four weeks after the intervention. An overview of participant flow and assessment points can be seen in Figure 1.

### Worry Postponement

Therapists were psychologists (M.Sc.) in advanced stages of their formal training to become licensed cognitive behavioral therapists. Treatment consisted of two sessions, one week apart, based on WP with a metacognitive rationale (Wells, 2004, 2007). Therapists followed a detailed manual which was adapted for this study to focus on the modification of uncontrollability beliefs by WP as a behavioral experiment. The first session (90 minutes) consisted of psychoeducation about worry and the diagnosis. A simplified individual case conceptualization was developed focusing on the belief that worry is uncontrollable, the subsequent attempts to control thoughts, and the resulting vicious cycle of worrying. Therapists used guided discovery techniques, hypothetical questions, and behavioral and thought experiments to socialize participants to the metacognitive model. WP was introduced as a behavioral experiment allowing to make new experiences with worrying and test uncontrollability beliefs. Specifically, participants were instructed to postpone worry throughout the day to a time frame not exceeding 30 minutes at a predefined time later in the day for the next six days. Participants were instructed to face worries throughout the day with an attitude of acceptance, to not get involved with the worry process, but to also not try to control worrying with previously used strategies. They composed a statement aimed at facilitating this endeavor (e.g., “Another worry arises, I acknowledge it, and now I let it go.”). The second session (60 minutes) focused on the evaluation of WP. After recapturing the insights of the first session, the experiences with WP were discussed. Participants were encouraged to devise new metacognitions and to adopt a new approach to managing worry (e.g., “I cannot control whether a worrisome thought comes to my mind, but I can control how I deal with it”).

### Measures

To assess negative metacognitive beliefs as our main outcome, we applied a subscale of the short German version of the Metacognitions Questionnaire (MCQ; Arndt et al., 2011). This self-report questionnaire is rated on a 4-point Likert-type scale ranging from “do not agree” to “agree very much”. For this study, we only used the negative beliefs about uncontrollability and danger subscale (MCQ-NEG), which consists of six items (example items: “My worrying thoughts persist‚ no matter how I try to stop them”, “My worrying is dangerous for me”). The MCQ-NEG has good psychometric properties (Arndt et al., 2011; Wells & Cartwright-Hatton, 2004); in the current sample the internal consistency at pre-assessment was Cronbach’s α = .77 for GAD and α = .86 for HYP.

Worry was assessed with the German version of the Penn State Worry Questionnaire (PSWQ; Stöber, 1995) with the instruction to rate the intensity of worry during the last seven days. The PSWQ consists of 16 items rated on a 5-point Likert-type scale ranging from “not typical of me” to “very typical of me” (example item: “I worry all the time”). The PSWQ has good psychometric properties (Fresco et al., 2002; Glöckner-Rist & Rist, 2014); the internal consistency was α = .73 for GAD and α = .86 for HYP at pre-assessment.

### Data Analyses

Our statistical approach was preregistered on the Open Science Framework prior to data analysis (see Krzikalla et al., 2022). Data were analyzed using the statistical processing language R (R Core Team, 2021). Missing data patterns were inspected with Little’s MCAR test (Little, 1988; χ^2^ = 453, *df* = 458, *p* = .556) and visual inspection. The assumption of a Missing at Random pattern was made due to the absence of contradictory evidence. Missing data on item-level was imputed by median-imputation. When post-assessment data was missing completely, we imputed by multiple imputation using predictive mean matching in the R package mice (van Buuren & Groothuis-Oudshoorn, 2011) to generate 20 imputed data sets. Auxiliary variables were identified using correlation analyses, univariate analyses of variance (ANOVA) and *t*-test comparisons (Enders, 2010). Statistical analyses were performed separately on each dataset and then pooled into a single set of results (Rubin, 1987). If not indicated otherwise, we report the results of the completer sample as results did not differ from the multiply imputed data (see Supplementary Materials).

Comparability of groups at baseline was analyzed by calculating independent *t*-tests for continuous variables and χ^2^-tests for categorical variables. Indicating partial failure of randomization, in the HYP group, the IG had significantly higher worry scores (PSWQ) at pre-assessment than WL (see Table 1). Next to the preregistered analyses, we added three further analyses to the Supplementary Materials of this article that take into account baseline differences.

To test the effect of the intervention, we calculated 2x2x2 mixed ANOVAs with the within-subjects factor *time* (pre/post), and the between-subjects factors *treatment group* (IG/WL) and *disorder* (GAD/HYP). Due to violations of homogeneity of variance, we calculated a robust ANOVA using the R package MANOVA.RM (Friedrich et al., 2019) and post-hoc Welch’s *t*-tests for MCQ-NEG. For PSWQ, we calculated a mixed ANOVA using the R package ez (Lawrence, 2016) and planned contrasts. In response to an inquiry of an anonymous reviewer, we performed a post-hoc power analysis using G*Power (Faul et al., 2007) for the Group*Time interaction. Based on previous research indicating small to medium effects of WP with a rationale of stimulus control on worry (Dippel et al., 2023) and large effect sizes for MCT (Normann & Morina, 2018), we calculated a power of .97 to find a medium effect (*f* = 0.25) with our total sample size of 80 participants. For a small effect (*f* = 0.1), our power would drop to .28.

To test the stability of the effect in the IG, we performed paired *t*-tests between pre-assessment and FU. For analyses with the FU data, we only used data of the IG as equivalence tests (Lakens, 2017) failed to indicate equivalence for the IG and WL at post-intervention. We calculated controlled effect sizes (Cohen’s *d* = (*m*_IG_-*m*_WL_)/*sd*_WL_; Cohen, 1988) for the pre-post-effect and uncontrolled effect sizes for the pre-FU-effect (Cohen’s *d* = (*m*_pre_-*m*_fu_)/*sd*_pooled_; Cohen, 1988). We assessed clinical significance according to Jacobson and Truax (1991) by using a reliable improvement criterion (RC = $\frac{x2-x1}{S\mathrm{diff}}$ ) and a recovery criterion (a = *M_1_* – 2*SD_1_).

## Results

### Sample Characteristics

**Figure 2:**
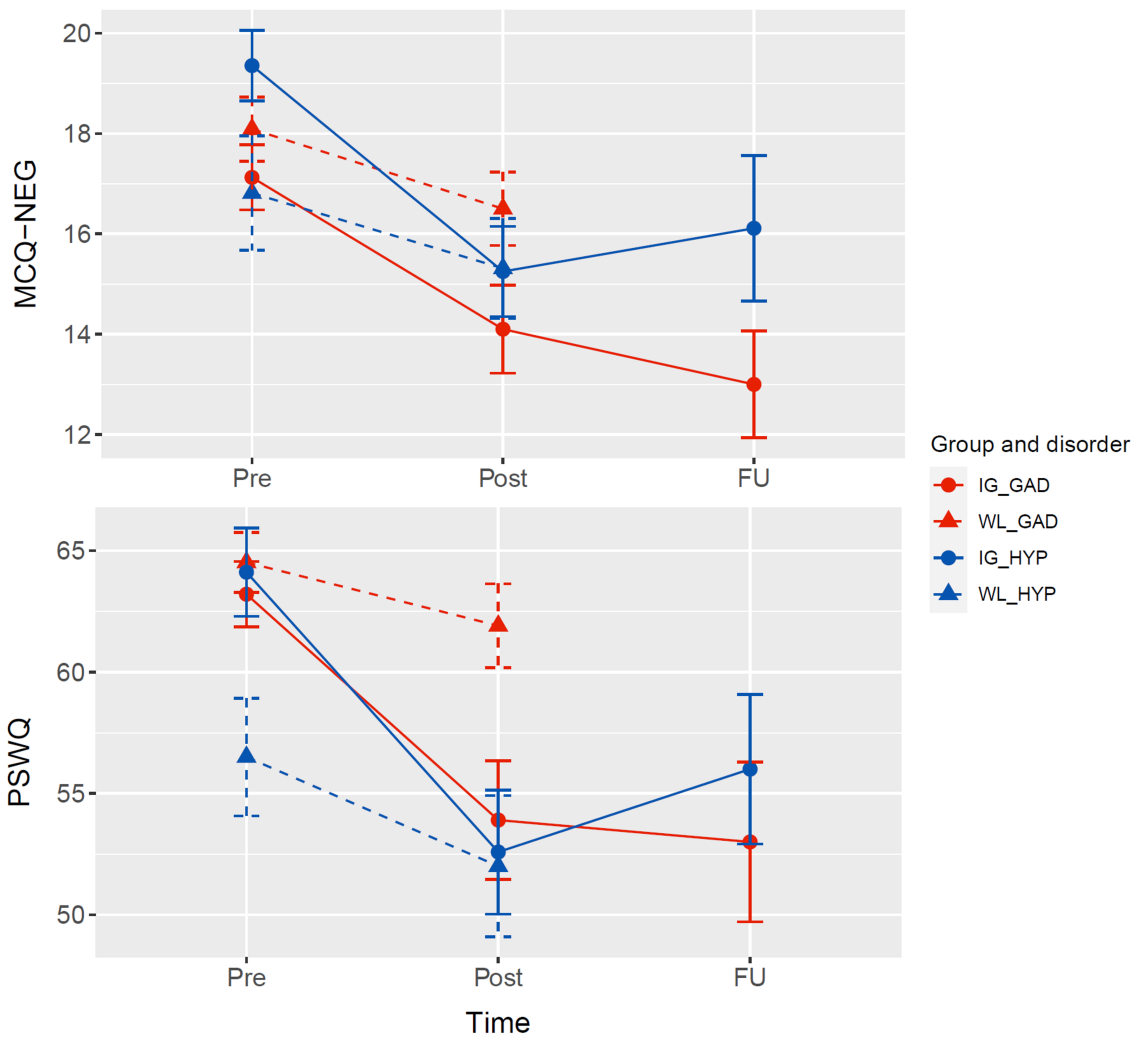
Change in MCQ-NEG and PSWQ Throughout Treatment *Note.* MCQ-NEG = negative metacognitions of the Metacognitions Questionnaire; PSWQ = Penn State Worry Questionnaire. Error bars represent standard deviation.

Table 1 gives an overview of demographic characteristics, comorbid diagnoses, and descriptive statistics along with tests of comparability of IG and WL at baseline separated for both clinical groups. The only significant difference between the IG and WL at baseline was in the HYP group, where participants in the IG reported higher scores on worry than participants in the WL (PSWQ pre). Figure 2 shows the descriptive statistics graphically.

**Table 1:** Characteristics of Participants and Descriptive Statistics Separated by Group and Disorder

Variable	GAD			HYP		
	IG	WL	*p*	IG	WL	*p*
**Age, *M* (*SD*)**	38.17 (13.21)	36.30 (14.36)	.646	32.18 (9.70)	36.81 (13.38)	.267
Sex, *n* (%)			.779			> .999
Female	18 (75.00)	19 (82.61)		11 (64.71)	10 (62.50)	
Male	6 (25.00)	4 (17.39)		6 (35.29)	6 (37.50)	
Marital status, *n* (%)			.663			.449
Single	5 (20.83)	3 (13.04)		3 (17.65)	1 (6.25)	
Partnered	11 (45.83)	10 (43.48)		7 (41.18)	6 (37.50)	
Married	8 (33.33)	9 (39.13)		7 (41.18)	9 (56.25)	
Divorced	–	–		–	–	
Widowed	–	1 (4.35)		–	–	
Highest educational level, *n* (%)			.649			.774
Non-academic high school	6 (25.00)	5 (21.74)		3 (17.65)	3 (18.75)	
Academic high school	9 (37.50)	9 (39.13)		6 (35.29)	5 (31.25)	
University or postgraduate degree	9 (37.50)	9 (39.13)		8 (47.06)	8 (50.00)	
Occupational status, *n* (%)			.640			.494
Full-time	10 (41.67)	8 (34.78)		4 (23.53)	4 (25.00)	
Part-time	5 (20.83)	4 (17.39)		4 (23.53)	4 (25.00)	
Retired	2 (8.33)	2 (8.70)		–	–	
Not-working	–	2 (8.70)		3 (17.65)	–	
Pupil/Student	6 (25.00)	7 (30.43)		5 (29.41)	6 (37.50)	
Other	1 (4.17)	–		1 (5.88)	2 (12.50)	
Comorbid diagnoses^a^, *n*			> .999			.616
Affective disorders	6	6		4	4	
Anxiety disorders (other than GAD)	4	5		4	4	
Obsessive-compulsive disorder	–	1		1	1	
Eating disorder	–	1		–	–	
Attention deficit hyperactivity disorder	1	–		–	–	
Adjustment disorder	0	–		–	1	
None	14	13		11	8	
MCQ-NEG pre, *M* (*SD*)	17.12 (3.18)	18.09 (3.06)	.296	19.35 (2.89)	16.81 (4.56)	.069
MCQ-NEG post, *M* (*SD*)	14.10 (3.92)	16.50 (3.43)		15.25 (3.11)	15.31 (3.98)	
MCQ-NEG follow-up, *M* (*SD*)	13.00 (4.39)			16.11 (4.34)		
PSWQ pre, *M* (*SD*)	63.21 (6.60)	64.52 (5.93)	.476	64.12 (7.50)	56.50 (9.71)	.018*
PSWQ post, *M* (*SD*)	53.90 (10.93)	61.91 (8.12)		52.58 (8.86)	52.00 (11.66)	
PSWQ follow-up, *M* (*SD*)	53.00 (13.58)			56.00 (9.25)		

*Note.* GAD = generalized anxiety disorder; HYP = hypochondriasis; IG = intervention group; WL = waitlist; MCQ-NEG = negative metacognitions of the Metacognitions Questionnaire; PSWQ = Penn State Worry Questionnaire; *p* = *p*-value for independent *t*-tests for continuous variables and χ^2^-tests for categorical variables for the difference between IG and WL. Significant *p*-values are marked in bold.

^a^Multiple responses possible, *p*-value relates to total number of participants with a comorbid diagnosis.

### Adherence and Competence Ratings and Credibility Check

Video tapes of both sessions for 16 participants (GAD *n* = 11, HYP *n* = 5) were randomly selected and rated by an advanced psychology student. Due to technical issues, only 14 videos could be included for the first session, 15 videos for the second session. Adherence and competence were scored on a 7-point Likert-type scale for each treatment element ranging from “none” (0) to “excellent” (6). Mean ratings of the total score for the first session were 5.90 (*SD* = 0.16) for adherence and 5.84 (*SD* = 0.31) for competence, for the second session 5.74 (*SD* = 0.37) and 5.73 (*SD* = 0.37) respectively. Four of the videotaped two-session-treatments were also rated by one of the coauthors (I.A. or J.W.). There were no relevant differences in the ratings.

Participants in the IG rated the credibility of the proposed intervention on a scale of 1 (“not at all”) to 10 (“very”) with three items at the end of the first session: rationale of the intervention (GAD: *M* = 7.26, *SD* = 1.91, *n* = 23; HYP: *M* = 8.47, *SD* = 1.55, *n* = 15), treatment benefit expectations (GAD: *M* = 5.87, *SD* = 2.10, *n* = 23; HYP: *M* = 6.73, *SD* = 1.75, *n* = 15), and if they would recommend it to a friend (GAD: *M* = 6.41, *SD* = 2.61, *n* = 22; HYP: *M* = 7.27, *SD* = 2.34, *n* = 15).

### Efficacy of Treatment

The results of the 2x2x2 mixed ANOVA for MCQ-NEG and PSWQ can be seen in Tables 2 and 3. The significant Time*Group interactions (*p* = .040 for MCQ-NEG and *p* = .003 for PSWQ) suggest a different effect of time in IG and WL.

**Table 2:** Results for the 2x2x2 Robust Mixed ANOVA for MCQ-NEG

Source of variation	*df1*	*df2*	*ATS*	*p*	$\eta_{G}^{\mathrm{2a}}$
Time	1	Inf	31.72	**< .001**	.09
Group	1	74.27	0.27	.605	.01
Disorder	1	74.27	0.06	.803	< .01
Time*Group	1	Inf	4.22	**.040**	.01
Time*Disorder	1	Inf	0.27	.605	< .01
Group*Disorder	1	74.27	3.06	.085	.03
Time*Group*Disorder	1	Inf	0.16	.685	< .01

*Note.* MCQ-NEG = negative metacognitions of the Metacognitions Questionnaire. Significant *p*-values are marked in bold.

^a^As there is currently no established protocol for obtaining $\eta_{G}^{2}$ after performing a robust ANOVA, the results after a mixed ANOVA are displayed.

**Table 3:** Results for the 2x2x2 Mixed ANOVA for PSWQ

Source of variation	*df1*	*df2*	*F*	*p*	$\eta_{G}^{2}$
Time	1	66	35.48	**< .001**	.11
Group	1	66	0.73	.396	.01
Disorder	1	66	7.14	**.009**	.08
Time*Group	1	66	9.19	**.003**	.03
Time*Disorder	1	66	0.98	.326	< .01
Group*Disorder	1	66	4.73	**.033**	.05
Time*Group*Disorder	1	66	0.02	.882	< .01

*Note.* PSWQ = Penn State Worry Questionnaire. Significant *p*-values are marked in bold.

We performed post-hoc Welch’s *t*-tests combined and also separately for the GAD and HYP group (Bonferroni-corrected α = .008, respectively) to analyze the significant Time*Group interaction for MCQ-NEG (Table 4). When analyzed across both diagnostic groups together, there was no significant effect (*t*(66.2) = -1.68, *p* = .049) after Bonferroni-correction. In the GAD group, the IG had significant lower scores in MCQ-NEG at post-assessment compared to the IG and WL at pre-assessment. In the HYP group, the IG had significantly higher scores at pre-assessment than the IG and WL at post-assessment. The other comparisons were not significant.

**Table 4:** Post Hoc Welch’s t-Tests for MCQ-NEG for the Total Sample and Separated for GAD and HYP

Comparison	Total sample			GAD			HYP		
	*df*	*t*	*p*	*df*	*t*	*p*	*df*	*t*	*p*
IG_post vs. WL_post	66.23	-1.68	.049	38.02	-2.10	.021	25.93	-0.05	.482
IG_pre vs. IG_post	31	4.31	**< .001**	19.00	2.64	**.008**	11.00	4.71	**< .001**
WL_pre vs. WL_post	37	2.95	**.006**	21.00	2.11	.047	15.00	2.01	.063
IG_pre vs. WL_pre	75.02	0.62	.538	45.00	-1.06	.296	25.12	1.90	.069
IG_pre vs. WL_post	73.91	2.63	.010	42.83	0.64	.526	27.30	3.32	**.003**
IG_post vs. WL_pre	67.09	-3.45	**< .001**	35.79	-3.68	**< .001**	25.82	-1.08	.146

*Note.* MCQ-NEG = negative metacognitive beliefs of the Metacognitions Questionnaire; GAD = generalized anxiety disorder; HYP = hypochondriasis; IG = intervention group; WL = waitlist. Alpha after Bonferroni-correction: α = .008. Significant *p*-values are marked in bold.

Concerning PSWQ, planned contrasts revealed that IG and WL differed significantly at post-assessment in the GAD group (*t*(101) = -2.95, *p* = .004), but not in the HYP group (*t*(101) = .17, *p* = .863). When analyzed across both diagnostic groups together, there was no significant effect (*t*(99.5) = -1.95, *p* = .054). We also observed significant main effects of Time in MCQ-NEG and PSWQ, and significant effects of Disorder and the Group*Disorder interaction for PSWQ. Post-hoc Welch’s *t*-tests to analyze the significant Group*Disorder interaction showed the only statistically significant difference in PSWQ between GAD and HYP in the waitlist groups (*t*(22.46) = 3.12, *p* = .005; further results in the Supplementary Materials).

Table 5 shows the controlled pre-post effect sizes, rates of response, deterioration, and recovery, separately for GAD and HYP. It also presents the results for the analysis of the FU data showing a significant reduction from pre to FU for MCQ-NEG in the GAD, but not the HYP sample, and a significant reduction in PSWQ for both samples.

**Table 5:** Effect Sizes, t-Test of Follow-Up Data, Response, Deterioration, and Recovery Rates for GAD and HYP

	Variable	Pre-post	RCI	Recovery criterion	Response, *n* (%)		Deterioration, *n* (%)		Recovery, *n* (%)		Pre-FU			
		*d* ^a^			IG	WL	IG	WL	IG	WL	*d* ^b^	*t*	*df*	*p*
**GAD**	**MCQ-NEG**	0.43 [-0.20; 1.06]	4.15	11.35	7 (35.00)	2 (9.09)	1 (5.00)	0 (0.00)	5 (26.32)	1 (4.77)	0.89 [0.16; 1.61]	2.95	16	.009
	**PSWQ**	0.82 [0.17; 1.47]	9.00	51.35	7 (35.00)	4 (18.08)	0 (0.00)	2 (9.09)	8 (40.00)	2 (9.09)	0.88 [0.22; 1.54]	3.19	16	.006
**HYP**	**MCQ-NEG**	0.67 [-0.14; 1.48]	4.10	10.22	4 (33.33)	1 (6.25)	0 (0.00)	1 (6.25)	0 (0.00)	1 (6.67)	0.63 [-0.34; 1.60]	1.51	8	.169
	**PSWQ**	0.76 [-.05; 1.57]	9.69	41.74	5 (41.67)	5 (31.25)	0 (0.00)	1 (6.25)	1 (5.00)	1 (6.67)	0.79 [0.00; 1.59]	2.43	8	.041

*Note.* GAD = generalized anxiety disorder; HYP = hypochondriasis; MCQ-NEG = negative metacognitive beliefs of the Metacognitions Questionnaire; PSWQ = Penn Worry Questionnaire; RCI = reliable change index; FU = follow-up; IG = intervention group; WL = waitlist; *d*^a^ = controlled effect size, Cohen’s *d*; *d*^b^ = uncontrolled effect size, Cohen’s *d*. For recovery, only participants who were over the cut-off at pre-assessment were evaluated.

## Discussion

To our knowledge, this is the first study that examines the efficacy of WP with a metacognitive rationale. The results support the efficacy of WP in reducing negative metacognitions and worry in participants diagnosed with GAD. However, for participants with HYP, the intervention demonstrated only limited efficacy.

We found differential effects over time between the IG and WL over both clinical groups. Subsequent post-hoc analyses of the significant Time*Group interaction for MCQ-NEG were barely non-significant for the total sample (*p* = .049) and the GAD sample (*p* = .021) after Bonferroni-correction (Bonferroni-corrected α = .008, see Table 4). However, there was a significant reduction of negative metacognitions from pre- to post-assessment in the IG of both clinical samples, which was not evident in the WL. Concerning worry, in the total sample, there was no significant difference between IG and WL (*p* = .054). In the GAD group, the IG had significantly lower scores at post-assessment than the WL. These results extend to the analysis of the follow-up data, where significant effects were observed from pre- to follow-up-assessment in the GAD group, demonstrating large effect sizes for negative metacognitions and worry. In the HYP group, only a significant reduction in worry was observed.

Thus far, evidence has indicated the efficacy of WP in non-clinical samples with low to moderate levels of worry. Contrary to the findings of the only study examining GAD patients (Tallon, 2019), in our study WP effectively reduced worry compared to a control condition in this population. Notably, all previous studies have employed a stimulus control rationale (Dippel et al., 2023; Tallon, 2019). Previous research demonstrated the efficacy of MCT (e.g., Normann & Morina, 2018) and of its components ATT and DM (Gkika & Wells, 2015; Knowles et al., 2016; Rochat et al., 2018; Rupp et al., 2019). Our results suggest that WP, as a behavioral experiment with a metacognitive rationale, is effective in reducing worry in GAD patients. This supports the metacognitive model regarding the importance of negative metacognitions in maintaining worry and the resulting potential to reduce worry by changing them (Wells, 2009). A tendency for these effects was also evident in the HYP group with reductions in metacognitions and worry in the IG but not in contrast to the WL. Previous research has shown an association between metacognitive beliefs and health anxiety (Keen et al., 2022) and preliminary evidence for the effect of MCT in hypochondriasis (Bailey & Wells, 2014). Changing uncontrollability beliefs may be less important in HYP. Another explanation for the lack of larger effects could be the failure of randomization, which resulted in the IG having significantly higher worry scores at pre-assessment than the WL.

Importantly, effects seem to be larger for worry than for negative metacognitions. This could indicate that even minor reductions in metacognitions have a significant impact on the extent of worries (change scores of the negative metacognitions and worry correlated with *r* = .54 across all participants). Alternatively, change of negative metacognitions might just represent one mechanism affected by WP. Even though WP mainly addresses the negative metacognition of uncontrollability (Wells, 2006), the intervention might also influence positive metacognitions. To a lesser extent, these are also linked to worry (Dugas & Koerner, 2005; Nordahl et al., 2023; Penney et al., 2013). Reduction of stimuli that trigger worry (Borkovec et al., 1983) or alterations of attentional control by training disengagement from worry (Hirsch & Mathews, 2012) might be further explanations for the reduction of worry after WP. Also, MCQ-NEG compromises the scale negative metacognitions from the MCQ (Wells & Cartwright-Hatton, 2004). This scale includes only three items addressing uncontrollability and three items addressing danger. The intervention did not explicitly target worry-related danger, which may have attenuated the effects. This is supported by the fact that while the intervention's effects are evident in the items related to uncontrollability, they are not observed in the items related to danger (Table S11 in the Supplementary Materials). In addition, we exploratorily analyzed a single item on the uncontrollability of worry at various points during the intervention, which also showed a significant decrease (see Appendix E in the Supplementary Materials).

It was not possible to combine the data of the IG and WL as planned due to lack of equivalence at post-intervention. The data suggest that this is not due to attrition, but rather that improvement occurred during the waiting period, resulting in a lower pre-intervention baseline of the WL. Tallon (2019) also reported a reduction of self-reported past-week worry over the course of their study in two different control groups. Besides regression to the mean, the repetitive measurements could have potentially altered attentional processes and shifted participants’ perspectives on their worries and thus the worries themselves.

To our knowledge, this is the first publication to address the efficacy of WP with a metacognitive rationale. Some limitations must be considered, when interpreting the results. We used questionnaires to measure negative metacognitions and worry, which may be biased. Additionally, uncontrollability of worries was assessed with the scale negative metacognitions of the MCQ, which also includes items concerning danger of worrisome thoughts. We did not include a measure of symptom severity for GAD or HYP, other than worry. Lastly, the results for the HYP sample must be interpreted with special caution as the sample size is somewhat small and pre-treatment difference in worries were evident. Further research, with an active control group, larger sample sizes, and the inclusion of additional mediators (e.g., positive metacognitions) is necessary to better understand mechanisms of change in WP.

### Conclusions

WP significantly reduced metacognitive beliefs of uncontrollability of worries and worry intensity in participants with GAD. However, a broader measure of negative metacognitions did not differ significantly between IG and WL. Further, we only found limited evidence for the efficacy in the HYP sample. Recovery rates of 40% in worry were achieved in the GAD sample, which is particularly remarkable given the short duration of the intervention. The study supports the application of WP with a metacognitive rationale for reducing negative metacognitions and worry, holding promise for further research and practical implementation.

## Supplementary Materials

The Supplementary Materials contain the following items:

- The pre-registration protocol for the study (see Krzikalla et al., 2022)
- Additional information (see Krzikalla et al., 2024):
  - *Appendix A:* Pooled results of the analyses with the multiply imputed data (Tables S1 to S6)
  - *Appendix B:* Additional analyses to validate the results relating to the failed randomization concerning the worry scores of the IG and WL in the HYP group at pre-assessment (Tables S7 to S9)
  - *Appendix C:* Post-hoc-*t*-tests for the significant Group*Disorder interaction in the mixed ANOVA for PSWQ (Table S10)
  - *Appendix D:* Results of the 2x2x2 robust ANOVA separately for the subitems of MCQ-NEG for uncontrollability and danger (Table S11)
  - *Appendix E:* Uncontrollability of worry measured with a single item (Tables S12 to S13)

KrzikallaC.
BuhlmannU.
SchugJ.
KopeiI.
GerlachA. L.
DoeblerP.
MorinaN.
AndorT.
 (2022). Supplementary materials to "Worry postponement from the metacognitive perspective: A randomized waitlist-controlled trial"
[Pre-registration protocol]. PsychOpen. 10.17605/OSF.IO/H48JW
PMC1130391539119056

KrzikallaC.
BuhlmannU.
SchugJ.
KopeiI.
GerlachA. L.
DoeblerP.
MorinaN.
AndorT.
 (2024). Supplementary materials to "Worry postponement from the metacognitive perspective: A randomized waitlist-controlled trial"
[Additional information]. PsychOpen. 10.23668/psycharchives.14458
PMC1130391539119056

## Data Availability

The data that support the findings of this study are available upon reasonable request from the psychotherapy outpatient unit of the University of Münster (contact via pta@uni-muenster.de). The data are not publicly available due to ethical restrictions.

## Reporting Guidelines

This article follows the guidelines of CONSORT statement.
